# Supplementary material for: Metabolic engineering of Methanothermobacter thermautotrophicus ΔH for recombinant acetoin production
Source: Metab Eng Commun. 2026 Mar 18;22:e00275. doi: 10.1016/j.mec.2026.e00275 (PMC13049647; doi:10.1016/j.mec.2026.e00275)
Supplement: Multimedia component 1 [file mmc1.docx]

**Supplementary material**

**Metabolic Engineering**

**Metabolic engineering of *Methanothermobacter thermautotrophicus* ΔH for recombinant acetoin production**

**Authors**

**Tina Baur^a^, Maximilliene T. Allaart^a,1^, Aaron Zipperle^a^, Gabriela Contreras^a^, Christian Fink^a^, Largus T. Angenent^a,b,c,d,e^, Bastian Molitor^a,b,f,*^**

^a^Environmental Biotechnology Group, Department of Geosciences, University of Tübingen, Schnarrenbergstraße 94-96, 72076 Tübingen, Germany

^b^Cluster of Excellence – Controlling Microbes to Fight Infections, University of Tübingen, Auf der Morgenstelle 28, 72074 Tübingen, Germany

^c^Max Planck Institute for Developmental Biology, Max Planck Ring 5, 72076 Tübingen, Germany

^d^Department of Biological and Chemical Engineering, Aarhus University, Gustav Wieds Vej 10D, 8000 Aarhus C, Denmark

^e^The Novo Nordisk Foundation CO_2_ Research Center (CORC), Aarhus University, Gustav Wieds Vej 10C, 8000 Aarhus C, Denmark

^f^Microbial Metabolic Biochemistry, Institute of Biochemistry, University of Leipzig, Johannisallee 21-23, 04103 Leipzig, Germany

* Corresponding author: Bastian Molitor, [bastian.molitor@uni-leipzig.de](mailto:bastian.molitor@uni-leipzig.de)

^1^ Present address: Center for Microbial Ecology and Technology (CMET), Department of Biotechnology, Ghent University, Ghent, Belgium; Center for Advanced Process Technology for Urban Resource recovery (CAPTURE), Ghent, Belgium


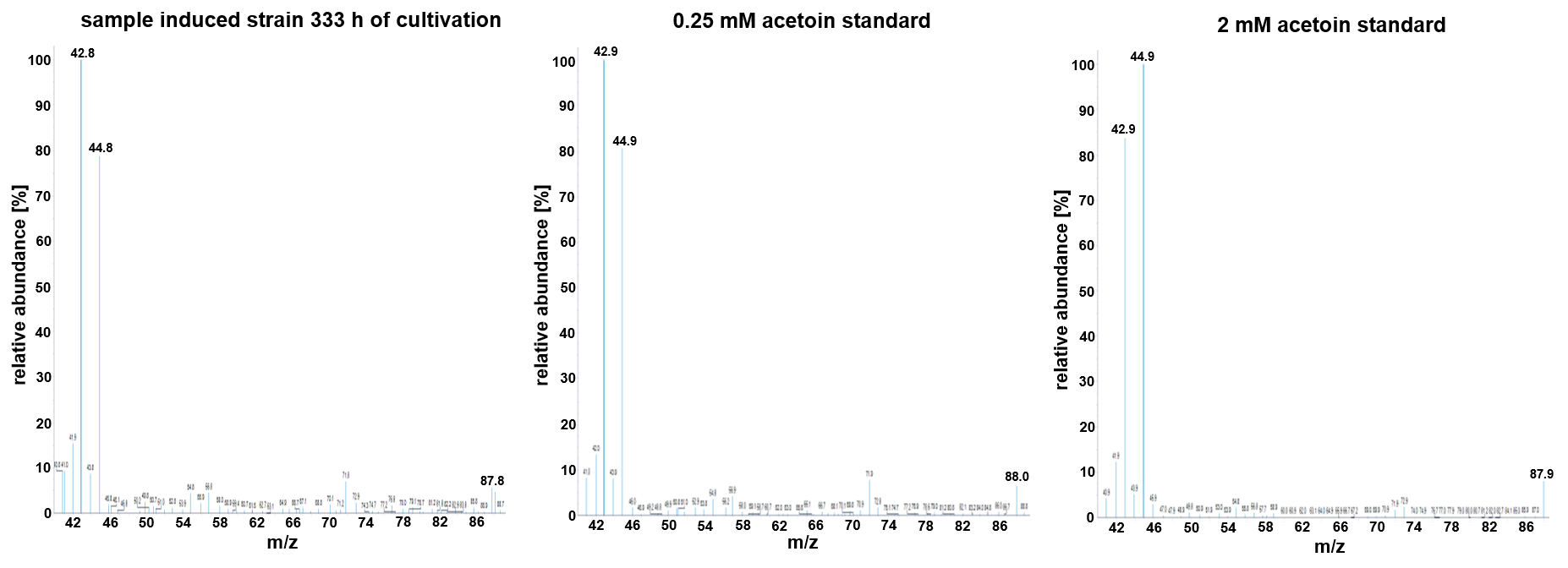


**A**

**B**

**C**

**Supplemental Figure 1. Mass spectra of chromatographic acetoin peaks of analyzed samples. A**, representative sample of induced *M. thermautotrophicus* [pMVS1111a_P*_tet_*_alsSD] culture (after 333 h of cultivation); **B**, defined 0.25 mM acetoin standard; **C**, defined 2 mM acetoin standard. A comparison of the spectra with the NIST database confirmed the identity of the compound as acetoin.


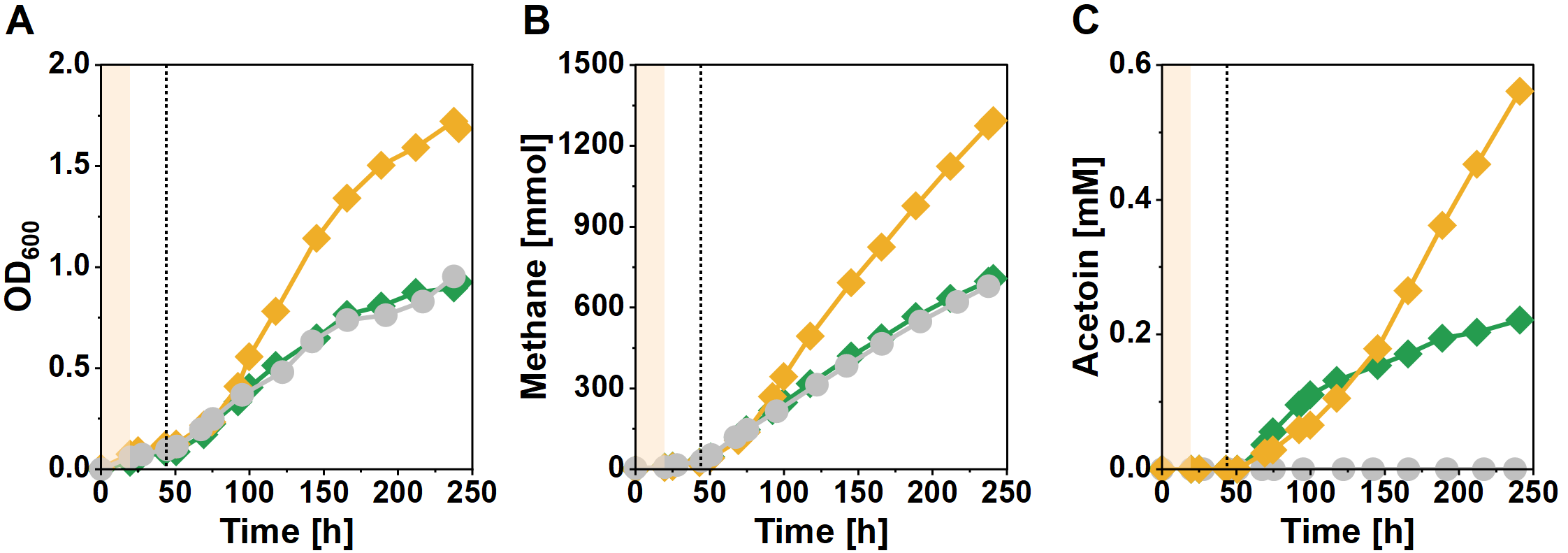


**Supplemental Figure 2. Recombinant acetoin production with *M. thermautotrophicus* in fed-batch bioreactors. A**, Growth (OD_600_); **B**, methane production; **C**, acetoin production. Grey circles, *M. thermautotrophicus* [pMVS-V1] (n=1); green diamonds, *M. thermautotrophicus* [pMVS1111a_P*_tet_*_alsSD] replicate 1; yellow diamonds, *M. thermautotrophicus* [pMVS1111a_P*_tet_*_alsSD] replicate 2. Initial growth occurred at 60°C (light orange shading), afterwards, the temperature was decreased to 42°C and acetoin production was induced using 1 µg mL^-1^ anhydrotetracycline (dotted line).


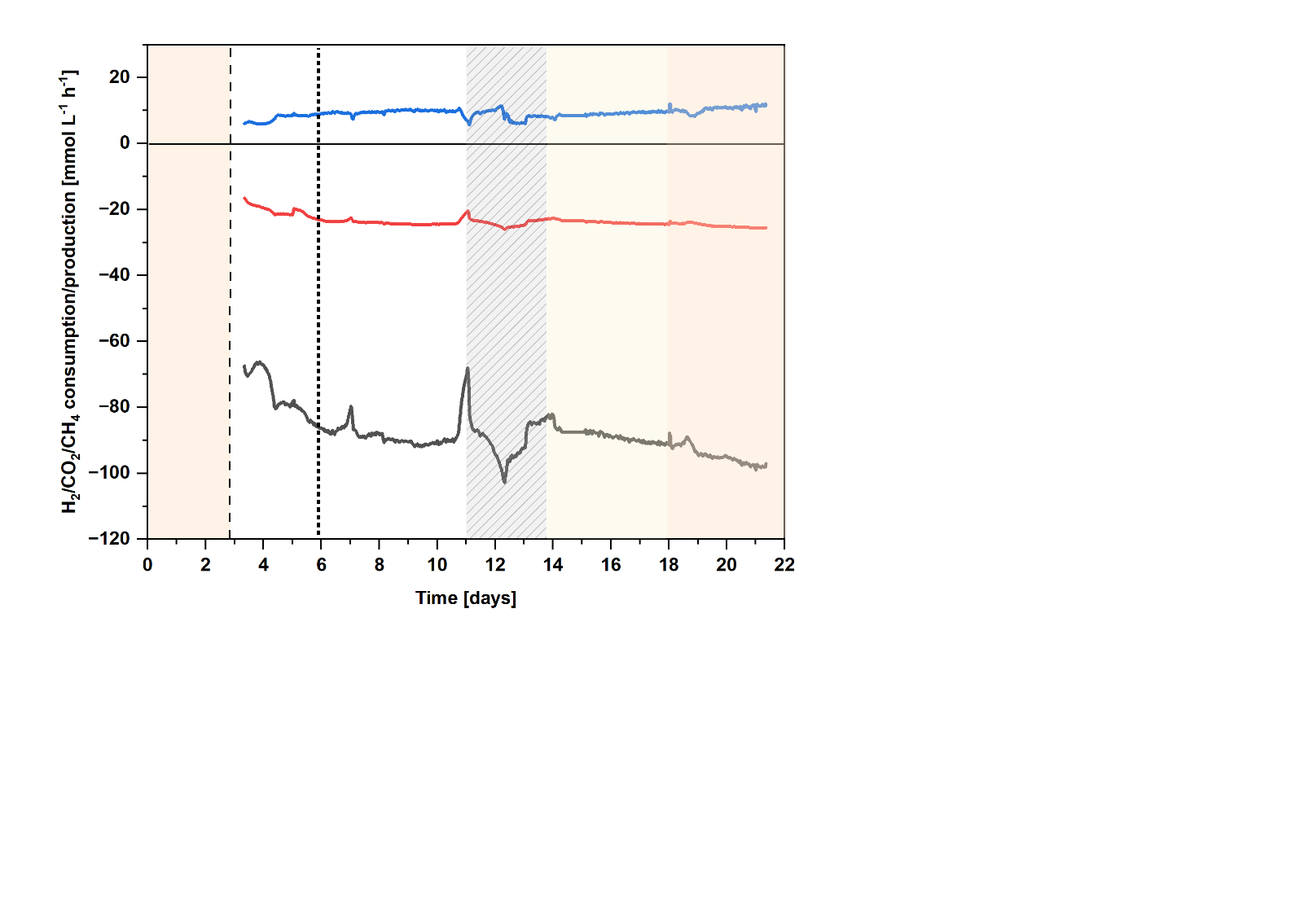


**Supplemental Figure 3.** **Gas data from chemostat bioreactor experiment related to Figure 5 in main text**. 5-point moving average of consumption- (grey, H_2_ and red, CO_2_) or production- (blue, CH_4_) rates are shown. For reference, acetoin production was induced with 1 µg mL^-1^ anhydrotetracycline (dotted line) after approximately 6 days. Light orange shading, cultivation temperature set to 60°C, light yellow shading, cultivation temperature set to 50°C; dashed line, switch from batch to continuous mode and decrease of cultivation temperature to 42°C; grey-hatched area, technical disturbances. n=1.

**Supplemental Table 1.** Sequences of codon-optimized *alsS* and *alsD* genes and gBlock fragments used for the construction of the recombinant acetoin-producing *M. thermautotrophicus* strain.

| Gene or gBlock | Sequence 5´→ 3´ |
| --- | --- |
| *alsS* | ATGAGCGAGGAGAAACAGCTGTACGGGGCCGACCTTGTAGTCGATAGTCTCATTAACCATGACGTAGAGTACGTCTTCGGGATACCGGGTGCAAAGATCGATAGGGTATTTGACACCCTTGAAGATAAGGGTCCAGAACTTATTGTGGCCAGGCATGAACAGAATGCAGCATTCATGGCCCAGGGGGTTGGAAGAATTACAGGAAAGCCAGGTGTAGTACTGGTAACCAGCGGACCTGGCGTCAGTAATCTTGCAACAGGGCTTGTGACAGCCACCGATGAGGGAGATCCGGTACTTGCTATTGGTGGACAGGTGAAGAGGGCTGACCTCCTTAAAAGGGCCCATCAGAGCATGAACAACGTTGCCATGCTCGAACCTATTACAAAGTATGCTGCTGAAGTACACGACGCCAATACACTGTCAGAAACCGTGGCCAATGCATATAGACACGCCAAGTCCGGTAAACCAGGTGCATCCTTTATCAGTATCCCGCAGGACGTTACAGATGCCCCGGTATCCGTCAAGGCAATCAAACCGATGACCGATCCGAAGCTTGGATCAGCTTCCGTATCCGATATAAATTACCTTGCACAGGCTATCAAAAATGCAGTTCTCCCAGTATTTCTGCTTGGGAATGGAGCATCATCCGAAGCCGTCACATATTCCATAAGACAGATCCTTAAACACGTAAAACTGCCGGTTGTCGAAACCTTTCAGGGCGCCGGAATTGTATCAAGAGACCTGGAGGAAGATACCTTCTTTGGCAGGGTCGGGCTCTTTAGAAATCAGCCGGGGGACATGCTCCTGAAGAAATCAGACCTGGTCATCGCCATAGGATACGACCCTATCGAATATGAGGCCAGGAACTGGAACGCCGAAATATCAGCCAGAATAATAGTTATAGACGTTGAGCCCGCAGAGGTCGACACCTATTTCCAGCCGGAAAGGGAGCTGATCGGGAACGTCGAAGCCAGTCTCAACCTTCTTCTCCCTGCCATACAGGGTTATAAGCTTCCAGAGGGCAGTGTTGAATATCTCAAGGGCCTCAAAAATAATGTCGTCGAAGATGTTAAATTCGATAGACAGCCGGACGAGGGAACAGTACATCCCCTTGATCTTATAGAAGTCCTTCAGGAACAGACCGACGATGACATGACCGTAACAGTGGACGTAGGCTCACACTATATATGGATGGCCAGGTACTTCAAGTCATACGAACCTAGACATCTCCTCTTTAGTAATGGAATGCAGACACTCGGAGTCGCTCTTCCCTGGGCTATATCAGCCGCCCTGGTAAGACCGAAAACAAAGGTTATCAGCGTTTCAGGAGATGGGGGCTTCCTTTTCTCCGCCCAGGAGCTCGAGACAGCAGTTAGACTTAAACTTCCGATCGTGCACATCATTTGGAACGACGGACATTATAACATGGTTGAGTTTCAGGAAGAGATGAAGTACGGTAGGTCCTCCGGAGTTGATTTCGGTCCTGTCGATTTCGTAAAGTACGCAGAAAGTTTTGGCGCTAAAGGCTACAGGGCTACCTCAAAGGCCGCATTTGCATCCCTGCTGCAGGAGGCTCTTACACAGGCAGTCGATGGTCCAGTGCTCATTGACGTTCCAATAGATTATAAAGATAACATAAAACTCGGGGAGACAATCCTGCCAGATGAATTTTACTAA |
| *alsD* | ATGAGCGAAGCTATTAAGCTTTTCCAGTATAACACCCTTGGGGCCCTTATGGCAGGCCTCTACGGCGGTACACTTACCGTAGGGGAACTGCTTGAACATGGCGATCTTGGACTCGGCACCCTCGATTCCATAGATGGTGAACTCATCGTACTGGACGGGAAGGCATACCAGGCTAAGGGCTCCGAAGGAAAAGTGGAAGTAGTCGAGGTTTCACCAGATGAAAAAGTTCCATATGCTGCCGTTGTCCCCCATCAGGCTGAAGTCATCTTTAGACAGAGATACGAAATGACCGATAAAGAGCTTGAGGACAGGATTGAGAGCTATTACGATGGCGTCAATCTGTTTAGAAGCATCAAAATCAAAGGTCACTTCAAGCATATGCACGTGAGAATGATACCCAAGTCAAATGCTGACATAAAATTTGCAGACGTGGCAACAAGACAGCCCGAATATGAGGTCGATGACATCTCCGGGACAATAGTAGGAATATGGACCCCGGAAATGTTTCACGGCGTTTCCGTGGCCGGGTACCATCTCCACTTTATCAGTGACGACCTCACCTTTGGCGGGCACGTTATGGACTTTGTCATTGAGAATGGTATCATCGAGGTAGGCCCTGTAGATCAGCTCGATCAGAGGTTTCCAGTTCAGGACAGACAGTACCTGTTCGCAAAGTTTAATGTCGATGAGATGAGGAAAGATATTACAAAGGCAGAATAATAA |
| gBlock tetracycline regulatory cassette | GGCGCGCCGGCCACGAGCTAAATTAAAGCGACTGCACTACTGTAAGGTCCGTCACGCACCATGAACCAACCGATGGCTCAGAAAAACCTTAAAATTAGCGATATATTTATATAGGATTATATGAATAGATAATATCACATAAAATGAGGTGGTTAATTATGTCAAGGCTGGACAAGTCAAAGGTAATCAACTCCGCCCTCGAGCTTCTCAACGAGGTGGGCATTGAGGGTCTCACCACAAGGAAACTTGCTCAGAAACTTGGGGTTGAGCAGCCCACACTCTATTGGCATGTCAAGAACAAGAGAGCCCTGCTTGACGCTCTGGCTATAGAAATGCTCGATAGGCATCACACCCACTTCTGTCCTCTCGAGGGGGAGTCATGGCAGGATTTCCTCAGAAATAATGCCAAAAGCTTCAGGTGCGCCCTGCTGTCCCACAGAGATGGTGCAAAGGTGCATCTGGGAACCAGGCCAACAGAAAAACAGTATGAGACCCTTGAAAACCAGCTTGCCTTTCTTTGCCAGCAGGGCTTCAGTCTGGAGAATGCACTCTACGCTCTGAGTGCCGTTGGTCACTTCACACTTGGATGTGTGCTCGAAGACCAGGAACATCAGGTTGCTAAAGAAGAAAGAGAGACCCCTACCACCGATTCCATGCCCCCGCTTCTCAGGCAGGCAATCGAGCTTTTTGATCATCAGGGTGCAGAGCCCGCCTTTCTCTTCGGTCTCGAGCTTATAATTTGTGGCCTGGAAAAACAGCTCAAATGTGAAAGTGGTTCATAGTAGGCCTCCAGTTCTCTTTTTCTTTTTTCTTTAACTTTACTTACTGCACTTTTATCCTCACTTTTTTCACTAGT |
| gBlock acetoin-production operon | ATGAGCGAGGAGAAACAGCTGTACGGGGCCGACCTTGTAGTCGATAGTCTCATTAACCATGACGTAGAGTACGTCTTCGGGATACCGGGTGCAAAGATCGATAGGGTATTTGACACCCTTGAAGATAAGGGTCCAGAACTTATTGTGGCCAGGCATGAACAGAATGCAGCATTCATGGCCCAGGGGGTTGGAAGAATTACAGGAAAGCCAGGTGTAGTACTGGTAACCAGCGGACCTGGCGTCAGTAATCTTGCAACAGGGCTTGTGACAGCCACCGATGAGGGAGATCCGGTACTTGCTATTGGTGGACAGGTGAAGAGGGCTGACCTCCTTAAAAGGGCCCATCAGAGCATGAACAACGTTGCCATGCTCGAACCTATTACAAAGTATGCTGCTGAAGTACACGACGCCAATACACTGTCAGAAACCGTGGCCAATGCATATAGACACGCCAAGTCCGGTAAACCAGGTGCATCCTTTATCAGTATCCCGCAGGACGTTACAGATGCCCCGGTATCCGTCAAGGCAATCAAACCGATGACCGATCCGAAGCTTGGATCAGCTTCCGTATCCGATATAAATTACCTTGCACAGGCTATCAAAAATGCAGTTCTCCCAGTATTTCTGCTTGGGAATGGAGCATCATCCGAAGCCGTCACATATTCCATAAGACAGATCCTTAAACACGTAAAACTGCCGGTTGTCGAAACCTTTCAGGGCGCCGGAATTGTATCAAGAGACCTGGAGGAAGATACCTTCTTTGGCAGGGTCGGGCTCTTTAGAAATCAGCCGGGGGACATGCTCCTGAAGAAATCAGACCTGGTCATCGCCATAGGATACGACCCTATCGAATATGAGGCCAGGAACTGGAACGCCGAAATATCAGCCAGAATAATAGTTATAGACGTTGAGCCCGCAGAGGTCGACACCTATTTCCAGCCGGAAAGGGAGCTGATCGGGAACGTCGAAGCCAGTCTCAACCTTCTTCTCCCTGCCATACAGGGTTATAAGCTTCCAGAGGGCAGTGTTGAATATCTCAAGGGCCTCAAAAATAATGTCGTCGAAGATGTTAAATTCGATAGACAGCCGGACGAGGGAACAGTACATCCCCTTGATCTTATAGAAGTCCTTCAGGAACAGACCGACGATGACATGACCGTAACAGTGGACGTAGGCTCACACTATATATGGATGGCCAGGTACTTCAAGTCATACGAACCTAGACATCTCCTCTTTAGTAATGGAATGCAGACACTCGGAGTCGCTCTTCCCTGGGCTATATCAGCCGCCCTGGTAAGACCGAAAACAAAGGTTATCAGCGTTTCAGGAGATGGGGGCTTCCTTTTCTCCGCCCAGGAGCTCGAGACAGCAGTTAGACTTAAACTTCCGATCGTGCACATCATTTGGAACGACGGACATTATAACATGGTTGAGTTTCAGGAAGAGATGAAGTACGGTAGGTCCTCCGGAGTTGATTTCGGTCCTGTCGATTTCGTAAAGTACGCAGAAAGTTTTGGCGCTAAAGGCTACAGGGCTACCTCAAAGGCCGCATTTGCATCCCTGCTGCAGGAGGCTCTTACACAGGCAGTCGATGGTCCAGTGCTCATTGACGTTCCAATAGATTATAAAGATAACATAAAACTCGGGGAGACAATCCTGCCAGATGAATTTTACTAAAAAGAGGGTGTAGACATGAGCGAAGCTATTAAGCTTTTCCAGTATAACACCCTTGGGGCCCTTATGGCAGGCCTCTACGGCGGTACACTTACCGTAGGGGAACTGCTTGAACATGGCGATCTTGGACTCGGCACCCTCGATTCCATAGATGGTGAACTCATCGTACTGGACGGGAAGGCATACCAGGCTAAGGGCTCCGAAGGAAAAGTGGAAGTAGTCGAGGTTTCACCAGATGAAAAAGTTCCATATGCTGCCGTTGTCCCCCATCAGGCTGAAGTCATCTTTAGACAGAGATACGAAATGACCGATAAAGAGCTTGAGGACAGGATTGAGAGCTATTACGATGGCGTCAATCTGTTTAGAAGCATCAAAATCAAAGGTCACTTCAAGCATATGCACGTGAGAATGATACCCAAGTCAAATGCTGACATAAAATTTGCAGACGTGGCAACAAGACAGCCCGAATATGAGGTCGATGACATCTCCGGGACAATAGTAGGAATATGGACCCCGGAAATGTTTCACGGCGTTTCCGTGGCCGGGTACCATCTCCACTTTATCAGTGACGACCTCACCTTTGGCGGGCACGTTATGGACTTTGTCATTGAGAATGGTATCATCGAGGTAGGCCCTGTAGATCAGCTCGATCAGAGGTTTCCAGTTCAGGACAGACAGTACCTGTTCGCAAAGTTTAATGTCGATGAGATGAGGAAAGATATTACAAAGGCAGAATAATAA |
